# Improving health and housing outcomes through a simulation and economic model: an evidence-based protocol of a group model building approach to develop an agent-based model

**DOI:** 10.3389/fpubh.2025.1623385

**Published:** 2025-08-01

**Authors:** Danielle M. Kline, Pranav Padmanabhan, Sarah E. Brewer, Magdalena Cerdá, Elysia Versen, Katherine M. Keyes, Margot Kushel, Erin C. Wilson, Paul Wesson, Ayaz Hyder, Alaina Boyer, Alia Al-Tayyib, Joshua A. Barocas

**Affiliations:** ^1^Division of General Internal Medicine, Department of Medicine, School of Medicine, University of Colorado, Aurora, CO, United States; ^2^Department of Epidemiology, Colorado School of Public Health, Aurora, CO, United States; ^3^Adult and Child Center for Outcomes Research and Delivery Science, University of Colorado, Aurora, CO, United States; ^4^Department of Family Medicine, School of Medicine, University of Colorado, Aurora, CO, United States; ^5^Department of Population Health, New York University Grossman School of Medicine, New York, NY, United States; ^6^Colorado Evaluation and Action Lab, University of Denver, Denver, CO, United States; ^7^School of Public Health, Columbia University Medical Center, New York, NY, United States; ^8^Division of Health Equity and Society, University of California, San Francisco, San Francisco, CA, United States; ^9^Center for Vulnerable Populations, University of California, San Francisco, San Francisco, CA, United States; ^10^Benioff Homeless and Housing Initiative, University of California, San Francisco, San Francisco, CA, United States; ^11^Public Health Foundation Enterprises, Inc., City of Industry, CA, United States; ^12^Department of Epidemiology and Biostatistics, University of California, San Francisco, San Francisco, CA, United States; ^13^Independent Consultant, DataToEngage, Futuwwa LLC, Columbus, OH, United States; ^14^National Health Care for the Homeless Council, Nashville, TN, United States; ^15^Public Health Institute at Denver Health, Denver, CO, United States; ^16^Division of Infectious Diseases, Department of Medicine, School of Medicine, University of Colorado, Aurora, CO, United States

**Keywords:** housing, homeless, group model building, agent-based model, HIV, substance use, overdose

## Abstract

**Introduction:**

Homelessness in the United States increased every year since 2016, with a 38% increase from 2023 to 2024. Much of the increase is attributable to rising home and rent costs, economic hardship caused by the recent pandemic, and the ending of protective legislation. Notably, people who experience homelessness have an increased risk of substance use disorders, HIV infection and poorer HIV outcomes than people who are stably housed. The iHouse model aims to develop feasible, effective, and cost-effective tailored approaches to improve health outcomes in this population including life expectancy, overdose, and HIV.

**Methods and analysis:**

The study will employ Group Model Building methods and use insights from that process to develop an agent-based model simulating the dynamic processes contributing to HIV incidence and treatment, overdose, and life expectancy among people along the housing and homelessness continuum in Denver, CO and San Francisco, CA. The model will evaluate multiple outcomes from 4 conceptual dimensions: (1) movement along the housing continuum, (2) population health (overdose and HIV incidence and life expectancy), (3) budgetary impact, (4) economic value.

**Ethics and dissemination:**

This study has been approved by the Colorado Institutional Review Board at the University of Colorado under protocol 24–0878. The data generated by this protocol, the methodologies used, and the findings will be made available in a timely manner to other researchers. iHOUSE code and parameter values will be published in Git Hub, such that all model analyses can be reproduced by independent investigators. Documentation of all parameter estimates and model results will be published for independent review and confirmation. In addition, supplemental materials and appendices for the model will be shared on a publicly available website.

## 1 Introduction

Homelessness in the United States (US) increased every year since 2016, with a 38% increase from 2023 to 2024 (1). Most recently, there were at least 771,480 people experiencing homelessness in the US on a given night—though these estimates only include people who were able to be found that night (2). The Department of Housing and Urban Development (HUD) defines homelessness as lacking a fixed, regular, and adequate nighttime residence (3). Housing instability is broader and includes several challenges, such as difficulty paying rent, overcrowding, moving frequently, or spending the bulk of income on housing (4). Much of the crisis is attributable to rising housing costs and rental prices, economic hardship resultant from the recent pandemic, end of protective legislation, and inaccessible services for people with chronic and stigmatized diseases that lead to poverty (5). Urban centers such as Denver and San Francisco have large populations of homeless people, but as housing costs continue to increase along with wage stagnation rural areas are at risk (6, 7).

Homelessness can lead to and is associated with profoundly negative health effects. People who experience homelessness (PEH) die on average 30 years earlier than other Americans. Notably, PEH have increased risk of substance use disorders and incident HIV than people who are stably housed. PEH with opioid use disorder (OUD) are less likely than their housed counterparts to receive treatment, and only 25% are receiving medications for opioid use disorder (MOUD) (8). Among people living with HIV (PWH), PEH were less likely to be engaged and retained in care and achieve virologic suppression (9).

Evidence-based interventions to improve health outcomes among PEH include both service- and housing-centered programs such as prevention and treatment services, transitional housing, voucher programs, and infrastructure development (10). These interventions are far from ubiquitously implemented. For example, only one in four eligible households receive assistance through the HUD’s Housing Choice Voucher program and wait times for the program vary widely by geographic area (11). Further complicating the issue, homelessness and health issues among PEH are heterogeneous, driven by locale-specific factors including service and housing availability and state and local housing policy. For example, while both San Francisco and Denver have sizable populations experiencing homelessness, the overlap with both OUD and HIV vary between these two locales. Questions remain regarding how to integrate and optimize service-delivery and housing-centered programs to improve health outcomes equitably for PEH under various scenarios.

To address this complex issue and provide policymakers with evidence upon which to base decision making, we plan a multiple methods approach that includes a Group Model Building (GMB) process and the design and implementation of an agent-based simulation model (12, 13). Specifically, we aim to develop the Improving Health Outcomes through a Simulation and Economic (iHOUSE) Model to simulate the dynamic processes contributing to overdoses, HIV, and life expectancy among people along the housing continuum of care in Denver and San Francisco.

Our aim is to use the iHOUSE model to simulate clinical and policy interventions at different points along the cascade. In the real world, such an approach would be cost prohibitive, unethical in many cases, and take years to produce results. Using an agent-based model, however, we can simulate large-scale randomized trials of interventions, in isolation and combination, providing rapid answers to critical policy questions. We can identify which policy “levers” can and should be pulled to optimize health outcomes in this population in a rigorous, randomized design. Ultimately, simulation models can quickly fill knowledge gaps and inform policy by serving as laboratories for testing hypotheses in real time.

We developed this protocol to provide an evidence base for the prioritization and optimization of strategies and structural interventions to improve health of PEH and decrease racial/ethnic health disparities in HIV, overdose, and life expectancy. This planned protocol assembles national leaders in community engaged research, housing and homelessness, HIV, substance use, mental health, and simulation modeling to develop a national resource that generates the scientific knowledge needed to improve health among PEH, reduce disparities, decrease overdose, and end the HIV Epidemic.

### 1.1 Overview

Our novel approach includes sequential components. First, we will develop a prototype of our agent-based model that utilizes the expertise of our study team as well as best practices for model building. Next, we will convene local stakeholders to inform priorities using a GMB approach. GMB is a participatory approach in which community stakeholders – including persons with lived experience, healthcare and public health providers, government officials, and national experts on homelessness and housing – collaborate to inform evidence-based models.

Third, we will use local data from public health partners to parameterize the model for both Denver and San Francisco, with the overall goal of informing policy by serving as a virtual, real-time laboratory to test the efficacy and sustainability of structural interventions to improve health outcomes for PEH. Once properly parameterized, we will have representative populations of people along the housing continuum in Denver and San Francisco and can then provide evidence on system innovations, projecting the population-level impact on health and costs by simulating individuals’ life courses under various scenarios (14).

### 1.2 Objectives

The first objective of the study is to employ a GMB approach to engage community stakeholders (a combination of government officials, private citizens, and direct service professionals, among others) in the scientific process and inform the development of agent-based models that simulate health outcomes along the housing continuum of care. The GMB process has been employed to develop system dynamics models and recently emerged as a tool for agent-based models. It is based on the idea that community members are experts in their own experience, thus, engaging them in the development of the evidence base can enhance the relevance of interventions, facilitate uptake and sustainability, and increase the likelihood of success (14). Ultimately, if we expect stakeholders to use a model to make or advocate for pragmatic decisions, they need to be involved from the beginning, so that they know what the options are, how the model works, and have a sense of ownership over the process. The GMB approach will enhance the model prototype, ensuring that relevant features are included to (1) capture the nuances of the housing continuum and (2) model pertinent policies and practical solutions.

We plan to engage community stakeholders in a process that will support implementation of this model as well as bridge divides between academic institutions and community. One key question for our stakeholder group is how to appropriately characterize the housing continuum and how individuals move within it. There are several different forms of being homeless (e.g., unsheltered, sheltered congregate, sheltered non-congregate) and different ways that people exit homelessness (supportive housing, subsidized housing, market rate housing). Our GMB group will ensure that the continuum that we model is representative of the continuum that exists both locally and nationally for individuals. Once we have a model prototype, we will engage in a GMB process to answer key questions about the model design and purpose. This will allow us to propose key model design attributes, calibration techniques, model outcomes, and a plan for setting up and testing experimental conditions.

The second objective is to develop agent-based models simulating the dynamic processes contributing to overdoses, HIV incidence and care engagement, and life expectancy among people along the housing continuum in Denver and San Francisco. We will use findings from the GMB to inform model structure and leverage unique local datasets to parameterize the models to develop representative populations of PEH in two cities – Denver and San Francisco. As previously mentioned, Denver and San Francisco are illustrative of the existing challenges across the US. Denver had a 30% surge in its homeless population within a year (6) and San Francisco has one of the nation’s highest rates of homelessness (7). The associated health challenges facing PEH differ between cities. These differences highlight the heterogeneous nature of homelessness in the US, indicating that a one-size-fits-all approach is inadequate. Thus, approaches that are informed by community are vital to improving health outcomes and ending homelessness. We will simulate cohorts of autonomous individuals who can interact with each other and their environment across time in accordance with behavioral rules developed from empirical evidence. With this we can estimate longitudinal health outcomes and economic costs in the current systems that serve PEH.

The third objective is to simulate and compare HIV and substance use service delivery programs implemented along the housing continuum versus housing-centered programs. We intend to answer the following research questions: 1. What are the changes in HIV incidence and overdose rates among the general population experiencing homelessness? 2. What are the relative potential impacts of these programs to address population-level needs and health outcomes? 3. How does service delivery along the housing continuum compare to housing-centered interventions in terms of HIV prevention and treatment, overdose reduction, and life expectancy improvements?

## 2 Methods and analytic plan

### 2.1 Design

#### 2.1.1 Study setting

Denver and San Francisco are ideal study locations because both cities have a high prevalence of homelessness and are experiencing increases in homelessness. For the purposes of GMB, both cities have strong partnerships between academic medical centers and community-based agencies, including local public health departments, and both have comprehensive linked databases that can facilitate a realistic, system-thinking simulation model. Additionally, while both are experiencing crises of homelessness and overdose, San Francisco is contending with high rates of HIV among PEH whereas Denver is not, thus we can compare different approaches in the two cities given their different health profiles. As such, we will be able to develop generalizable messages for jurisdictions with and without high HIV incidence by conducting sensitivity analyses to identify the factors that drive findings and forecast how jurisdictions with different sociopolitical, geographic, and epidemiologic profiles may have different priorities.

#### 2.1.2 Group model building

We will employ a GMB approach to inform the development of the simulation models. The GMB process will take place with two key groups. First, the Core Modeling Team will be convened. The Core Modeling Team will comprise 5–8 members and will include research team members and community leaders with subject matter expertise to guide the overall GMB process. They will be responsible for defining and recruiting workshop participants, developing the key questions and activities for workshops, and for assisting the research team with interpreting workshop results to be incorporated in the agent-based model. The Core Modeling Team will also inform the format and duration of workshops, including the length of each session (e.g., full day, half day, multi day), the number of workshop sessions needed, frequency of workshops, and if there is a need to include additional stakeholders in the process. Second, we will convene GMB workshop participants who will iteratively develop the final causal loop diagram across a series of group workshops.

We will recruit, via snowball sampling, policymakers (e.g., local public health department personnel and government officials), community-based experts, and people with lived experience to serve as the GMB Core. We will email or call the selected participants to request participation with the details of the research project and the GMB process. We plan to recruit people from each city to engage in the GMB process. We will employ broad recruitment strategies and seek to engage a varied set of GMB workshop participants who either work with individuals or have lived experience with homelessness.

We will use best practices for using GMB for system dynamics modeling and apply it to our agent-based model process. It will involve a series of structured small group discussions that illuminate the socio-cultural context and the issues that need to be addressed. We will use a modified concept mapping framework for this process, which our team has specific expertise in applying (15).

We anticipate holding 3–5 GMB workshop sessions with participants with the explicit purpose of augmenting and/or adjusting our preliminary model to investigate the interactions of people along the housing continuum with each other, services, and their environment, and how each may impact health. We will supply a reference model (i.e., prototype) to stakeholders as opposed to a blank piece of paper. This allows for open dialog about what is wrong with our conception of the problem and the structure of the model. During the first GMB workshop, we will present the reference model including the agents and state variables and the framework for interactions between agents that would lead to emergent patterns at higher scales to develop an agent-based model. Over the sessions, the group will have the opportunity to reshape these model components. Following the approach by Koh et al., the objectives of the first GMB workshop(s) will be to (1): define the problem (2), refine the model boundaries, and (3) augment/change an estimable model that captures core structures of the system. During the subsequent GMB workshop, participants will discuss the model outputs (and their appropriateness), clarify simulated behaviors of agents, and provide face validation of the model’s inner workings (12). The objectives of the later GMB workshops will be to (1): discuss relevant strategies and policy options (2), review the agent-based model and suggest improvements, and (3) identify future collaborations among the participants using the final agent-based model.

##### 2.1.2.1 GMB Workshop topics

###### 2.1.2.1.1 Identifying main barriers

The objective of this exercise will be to identify barriers that prevent individuals from moving upwards along the housing continuum. This exercise will help us confirm and/or adjust a priority list of variables, processes, and agents to be included in the model. The GMB participants will be asked the following question: “What are the barriers that prevent individuals from moving along the housing continuum toward stable, long-term housing?” The study team will then compile a consolidated list of factors to remove duplicates and combine analogous ideas. Once the list is compiled, participants will be asked to sort the factors into piles that they deem to be similar in the context of the prompt and instruct them to name each pile. Once factors are sorted, participants will rate each factor on a 5-point Likert scale relating to (1) how much each factor relates to housing instability/homelessness and (2) how common it is.

###### 2.1.2.1.2 Mapping interactions

At the beginning of the session, the reference model will be presented. Participants will be asked to create their own conceptual model by mapping the interactions between important factors leading to housing instability and homelessness (or leading people out of it). The structure of the computational model will be iteratively adjusted based on participant feedback. This may include theoretical transitions between housing states, influences of housing variables on health states, or key interactions between individuals and providers.

###### 2.1.2.1.3 Key stakeholders

Finally, participants will be asked to identify key stakeholders—who might be best fit to address the barriers identified earlier. Each stakeholder identified will be placed on a horizontal-vertical plane indicating levels of interest and power (low to high), respectively.

###### 2.1.2.1.4 Intervention levers

The aim of this exercise will be to identify intervention levers that could be implemented to improve health outcomes and move people along the housing continuum. Participants will propose interventions and others will be able to vote for the most important levers. Proposed interventions will directly inform model parameter selection.

Parameter estimation: Based on the initial structure of the model after the mapping interactions activity, participants will be asked to make a list of model parameters, identify known or potential data sources for each model parameter, and, if data sources are unknown, then estimate based on their professional expertise the range of values for model parameters that make sense to them. The output from this activity will inform the model calibration process for the agent-based model.

###### 2.1.2.1.5 Validating models

The objective will be to elicit feedback from participants on the near-final version of the model. This will be done by presenting the near-final model to the group and explaining how the modeling team incorporated the conceptual models designed by the GMB participants at the first workshop into the model. Participants will be divided into groups to further elucidate any additional changes to the model, the intervention levers, or the structure of the model.

##### 2.1.2.2 Modeling

Simulation modeling is an iterative process in which a conceptual model is used to plan and execute the computational model which is informed by community insight. This is particularly true for this project since we will be using insights from the GMB process to inform the final structure of the model (16–21). We put forth here a preliminary structure for this agent-based model that we expect to transform through the GMB process.

###### 2.1.2.2.1 Model conceptualization

Following the best practices for conceptualizing a model (22), we chose an agent-based model. Agent-based modeling is a “computational approach in which agents with a specified set of characteristics interact with each other and with their environment according to predefined rules.” (23–25) This means that we can model events occurring to and policies acting on individuals over time, and how the individual interacts with other people, resources available, and the larger environment during that time. In agent-based models, geographic and environmental conditions such as weather can be included.

###### 2.1.2.2.2 Focus on the housing continuum, service delivery, and housing programs

**Figure 1:**
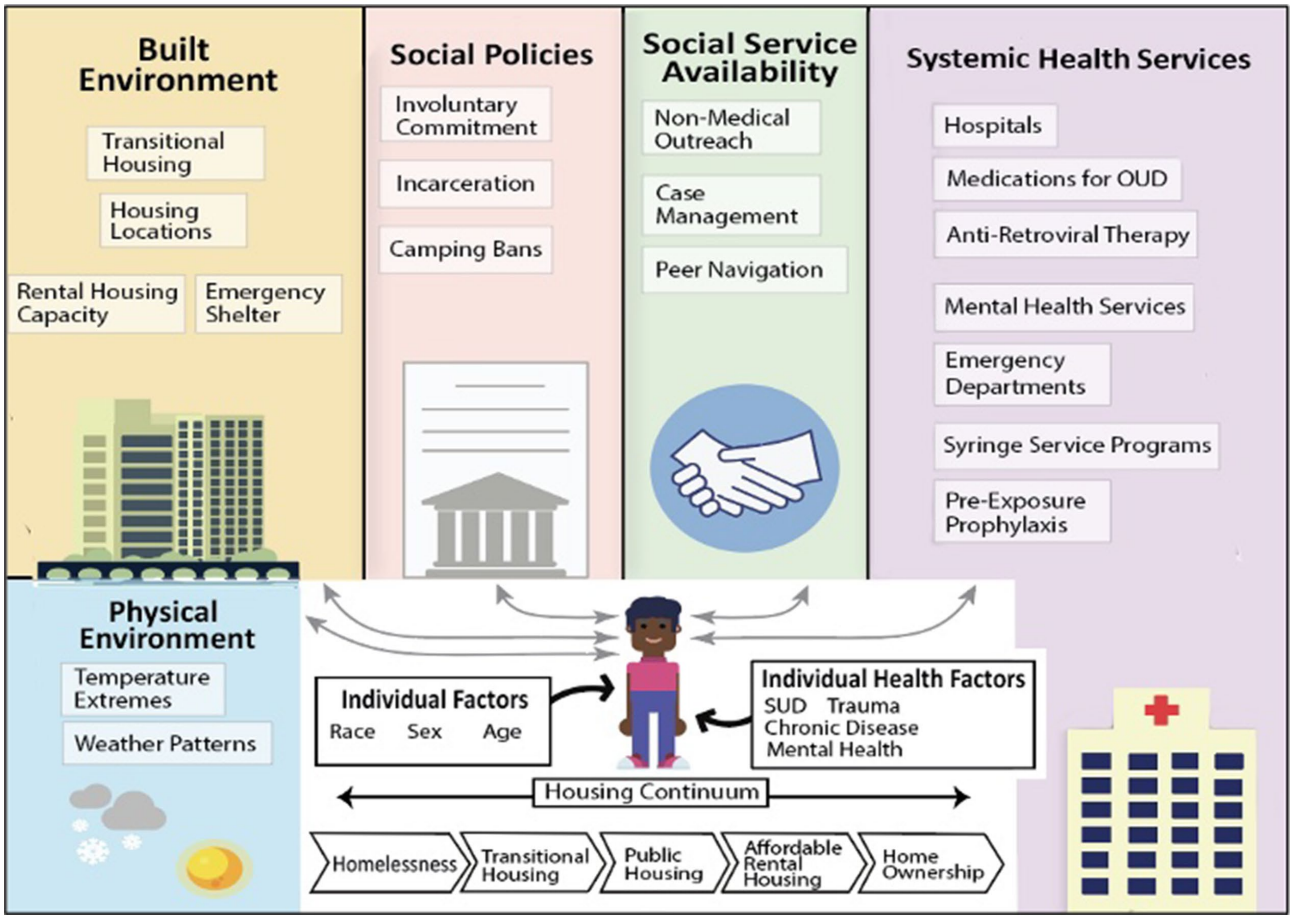
Example illustration of the potential components that influence health outcomes of agents in the iHOUSE Model.

Our model will focus on the housing continuum of care. Though the housing continuum can look slightly different by jurisdiction, the conceptual model from which they are all drawn includes homelessness (unsheltered and sheltered in congregate or non-congregate settings), transitional housing, public and affordable rental housing, market rental housing, and homeownership (Figure 1). Given that there is deleterious health effects associated with unstable housing and homelessness compared to stable housing, we will adapt this continuum such that the focus is on homelessness, transitional housing, public housing, affordable rental housing, and homeownership.

The model will allow simulated people (“agents”) to move along the continuum. Furthermore, it will be structured such that a cadre of services may be delivered to directly improve other major health outcomes at various steps along the continuum (e.g., PrEP, naloxone distribution, MOUD). Inclusion of both housing status and service delivery as influencing parameters is important since evidence suggests that individuals should have access to low barrier services that they are ready to accept, regardless of their other circumstances (i.e., where they live, what their drug use looks like). We will be able to use the model to compare, for example, the effectiveness of PrEP as an intervention for HIV prevention delivered at various points along the continuum as well as the comparative effectiveness of PrEP versus moving people into more stable housing. As such, this will allow stakeholders to determine where the greatest efficiencies might be in preventing HIV, reducing overdose, and improving life expectancy for people wherever they are on the continuum. We will be able to understand which strategies may have the greatest effect at reducing HIV and overdose in each city – thus, allowing stakeholders to tailor interventions to the local context.

###### 2.1.2.2.3 Model structure

The agent-based model (ABM) we are developing is intended to simulate transitions along the homelessness and housing continuum in an urban context. Because the final model structure will be guided by ongoing data acquisition, stakeholder input, and iterative calibration, we describe here the preliminary conceptual approach.

##### 2.1.2.3 Population creation

We anticipate generating a synthetic population of agents representing individuals who are at risk for or currently experiencing homelessness. Preliminary plans include assigning agents demographic, health, and service-use attributes based on distributions drawn from linked administrative datasets (e.g., HMIS, behavioral health service data, public health surveillance). Agent characteristics will include age, sex, race/ethnicity, HIV status, substance use history, and housing history, among others. The size and composition of the synthetic population will be determined during model calibration to reflect the characteristics of the study city at model initiation.

##### 2.1.2.4 Spatial structure

We expect to create a simplified spatial structure that captures key locations relevant to the homelessness and housing continuum, such as emergency shelters, transitional housing, permanent supportive housing, public spaces, and healthcare facilities. Spatial organization may be represented as zones or coordinate-based locations, depending on data availability and computational considerations. Final decisions about spatial resolution will be made after preliminary testing and in consultation with subject matter experts.

##### 2.1.2.5 Agent connections

We intend to incorporate dynamic social networks among agents to capture formal and informal ties, including co-residence in shelters, service participation, and peer relationships. Social connections will likely be probabilistic and influenced by factors such as demographic similarity, shared experiences, and physical proximity. The structure and strength of connections will be further refined as model parameterization proceeds.

##### 2.1.2.6 Behavioral rules for interaction and action

Agent behaviors will initially be governed by rules describing movement across housing states, utilization of services, and health-related behaviors such as engagement with HIV or substance use treatment. Interaction rules may allow agents to influence each other’s housing trajectories or service engagement, although the exact mechanisms will be determined based on data analysis and model calibration. Behavioral transitions will include stochastic elements and will be informed, where possible, by empirical transition probabilities derived from linked datasets. Transition probabilities for housing and health states (e.g., transitions between unsheltered homelessness and congregate shelter, non-congregate shelter, or supportive housing, and between HIV disease states) will be dependent on an individual’s characteristics and health behaviors, and will be derived using multinomial logistic regression equations.

**Figure 2:**
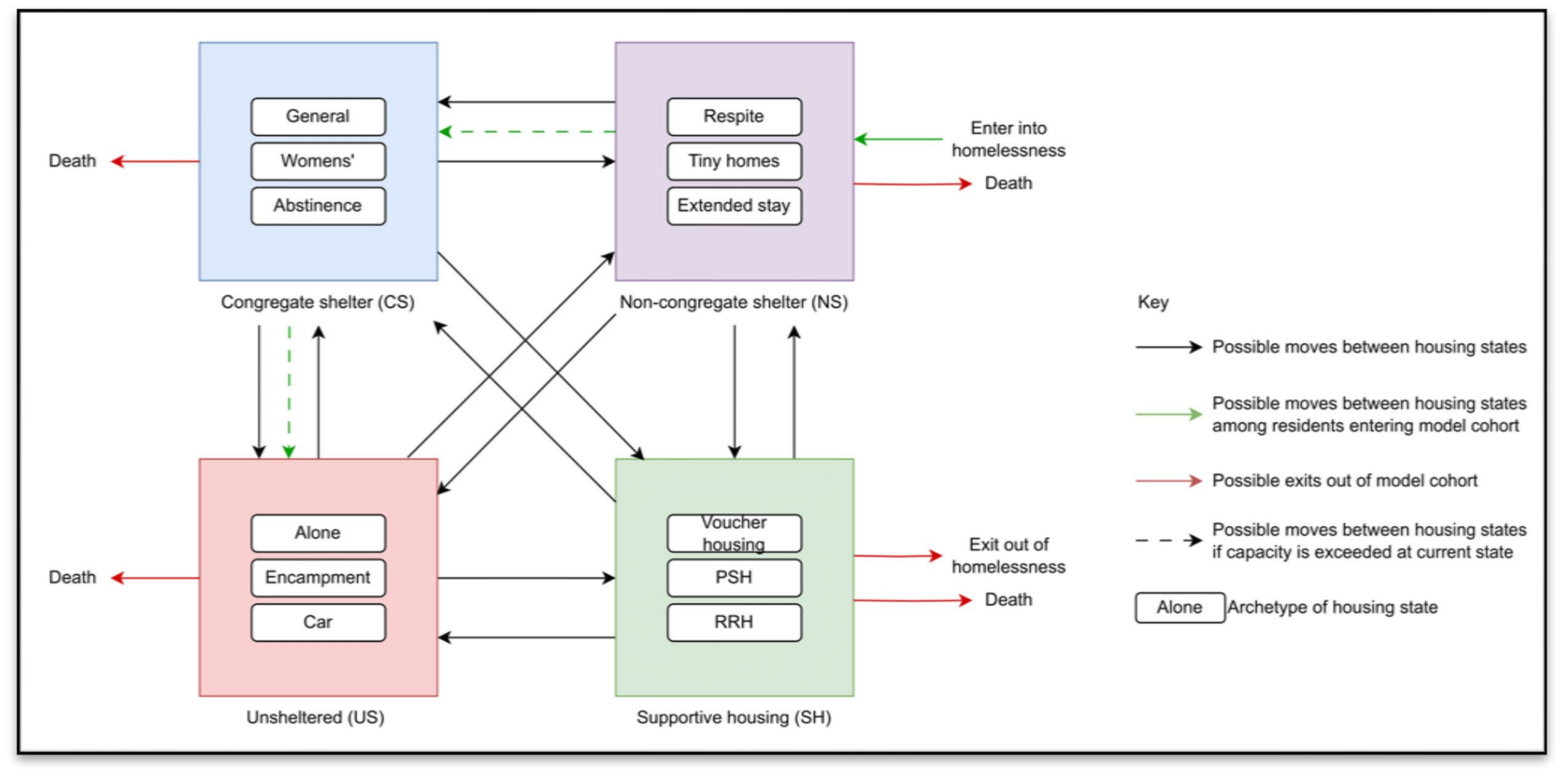
Conceptual framework of housing states and transitions for agent-based model of the spectrum of housing insecurity.

Given that the model is still in active development, these structures will be iteratively refined in response to emerging data, expert input, and model calibration needs. As an example, Figure 2 depicts a conceptual framework of housing states and transisitions for an agent-based model of the spectrum of housing insecurity. Key assumptions and parameter choices will be transparently documented as part of the final model specification.

After specifying the model structure, we will ensure that the data are properly calibrated to historic city-specific data and validate it by seeking input from other experts in the field and GMB participants. Once final adjustments are made to the structure, we will validate and calibrate the model. To formally validate the model, we will rely on established best practices and agent-based modeling procedures. Bayesian emulators or machine learning techniques may be used for model calibration (26). After specifying initial conditions, parameter values, and the agent interactions, we will conduct extensive sensitivity analyses, including parameters sweeps and Bayesian melding techniques, to test model robustness and express uncertainty about outputs. We will use empiric data (e.g., overdose and other unnatural death incidence estimates) to calibrate the models, and goodness of fit will be assessed with established methods (26). Since the systems being analyzed include many stochastic processes, we will conduct Markov Chain Monte Carlo simulations to generate distributions of outcomes. Simulations are computationally intensive; thus, the model will likely run on a high-performance supercomputing cluster located at the University of Colorado (27, 28). To manage complexity in case of unwieldy runtimes, the model will be developed flexibly such that certain interactions and functions may be disabled for given analyses depending on the emphasis of the research question. We will consider trade-offs between granularity and runtime during model development, which will be informed by ongoing iterative runtime testing of model components.

###### 2.1.2.6.1 Key components of the ABM

**Progression along the housing continuum**: The core of the simulation will be a state-transition model of housing instability and homelessness with mutually exclusive states that are likely to include unsheltered homelessness, congregate shelters, non-congregate shelters that include low and high barrier programs, transitional housing, supportive housing, unsubsidized rental housing, and homeownership. Agents will “leave” the model if they die. New agents enter the model at age 18. Transitions between states will be a function of local demographic characteristics, as well as selected health conditions, interactions between agents, natural and built conditions, and service availability. Where an individual is on the continuum will influence HIV and overdose risk, mortality rates, quality of life (defined in terms of health state utility), and service utilization and other costs.

**Physical environment**: Certain aspects of the physical environment (i.e., natural environment) can influence the health outcomes of individuals with housing instability. Specifically, we will include temperature extremes and snowfall that may impact an individual’s mortality risk (29).

**Systemic health services**: We will include engagement with health services as treatment episodes including MOUD, antiretroviral treatment (ART), and PrEP, as well as hospitals, emergency departments, and mental health services. While engaged with a treatment episode, individuals may or may not be actively using drugs or engaging in other behaviors that put them at risk for HIV. Health care service (MOUD and mental health services) episodes can affect movement along the housing continuum, as well as mortality. We will model at least two health service types: (1) HIV-related prevention or treatment and (2) drug use-related treatment (MOUD), with the possibility of including mental health services and primary care.

**Social service availability**: We will include social services including non-medical outreach and housing services. Interactions with each of these will differentially influence the probability of an individual’s uptake of and retention in health care services.

**Social policies**: Certain aspects of the social environment-specifically, municipal policies-can influence the health outcomes of individuals with housing instability. Some local policies (both supportive and punitive) can alter an individual’s probability of seeking shelter, services, and HIV and overdose risk behaviors.

**Built environment**: Movement along the housing continuum will be dependent on shelter and housing capacity. For the purposes of this model, the built environment will include emergency shelter (with differences between Denver and San Francisco in the types of emergency shelter), transitional housing, and rental housing capacity. We will use publicly available data to spatially place available housing services within the environments of each city. We will use locally specific plans (e.g., the Denver Mayoral plan for housing expansion) to test how changes to the built environment may influence agents and impact outcomes.

**Costs**: The model will estimate costs from multiple perspectives, including societal, healthcare sector, relevant state budget components (e.g., Medicaid), commercial payers, and service providers.

##### 2.1.2.7 iHouse

We will use the virtual, real-time laboratory to move research into action. When it comes to the overlapping epidemics of homelessness, HIV, and overdose, there are several evidence-based interventions. Housing First models have demonstrated improved outcomes regarding healthcare utilization and criminal legal involvement (30, 31). Moving people into more stable housing that meets their needs has been shown to decrease infections and reduce unsafe drug use. At the same time, the number of efficacious interventions to prevent and treat substance use and HIV continues to grow, including low barrier MOUD (32, 33), procurement of clean injection equipment, and PrEP (34). One barrier to implementation is policymakers not knowing what to do first, and how to pay for it.

The question of when, for whom, and in what context do housing-centered approaches improve health outcomes and are cost-effective compared to service-centered approaches is unknown—a gap in knowledge that we plan to fill with these analyses.

### 2.2 Data

In simulation modeling, the robustness and fidelity of outcomes are tethered to the quality and comprehensiveness of the underlying data. Input parameters can be classified as “acontextual” or “contextual.” Acontextual parameters are those that, in general, do not differ based on geographic or demographic characteristics. These parameters will be largely informed by published literature. Examples of acontextual parameters include HIV disease progression or the efficacy of MOUDs. There is an abundance of published literature for these “acontextual” parameters. Context, however, may influence certain parameters such as uptake of MOUD or time to HIV diagnosis. Aside from published studies, input parameters for the base model will be drawn from three primary sources:

1. For the Denver parameters, we have partnered with the Colorado Lab at the University of Denver where the Linked Information Network of Colorado (LINC) program is run. LINC is a collaborative based out of the Governor’s Office of Information Technology that supports timely and cost-efficient research, evaluation, and analytics. LINC can integrate data across state and local agencies including human services, health, labor and employment, higher education, housing, K-12 education, and criminal justice. Data are linked at the individual-level across databases from state government agencies (Table 1).
2. For the San Francisco parameters, we plan to use San Francisco’s Coordinated Case Management System (CCMS) in collaboration with the Benioff Homelessness and Housing Initiative at UCSF. Much like LINC, CCMS links San Francisco Department of Public Health (SFDPH) data, with San Francisco Medicaid, jail, substance use and mental health systems, emergency medical services, and housing and homelessness data at the individual-level to answer policy-relevant questions.
3. We plan to collaborate with local National HIV Behavioral Surveillance (NHBS) projects to augment city-specific data when needed. NHBS is a comprehensive system for bio-behavioral surveillance conducted since 2003 in populations with high burden of HIV. NHBS collects data on behavioral risk factors for HIV (e.g., sexual behaviors, drug use), HIV testing behaviors, receipt of prevention services, and use of prevention strategies (e.g., PrEP). Both Denver and San Francisco are NHBS sites, and both have included comprehensive questions regarding HIV and overdose risk among PEH in the 2022 and 2024 cycles, as well as homelessness specific questions.

**Table 1:** Preliminary parameter list for iHOUSE.

Parameter	Description	Potential data source(s)
Initial population size	Number of agents at model start	HMIS point-in-time (PIT) count; Local administrative homelessness data
Initial housing status distribution*	% unsheltered, sheltered, transitional housing, supportive housing, housed with voucher, market rental, homeownership	HMIS annual homeless assessment report (AHAR); Local continuum of care (CoC) reports
Demographic distributions	Age, sex, race/ethnicity breakdowns among agents	HMIS data; behavioral health administration (BHA) datasets; DDPHE HIV surveillance
HIV prevalence	% of population with HIV	DDPHE HIV surveillance; CDC HIV surveillance report; Local health department data
Substance use disorder (SUD) prevalence*	% of population with opioid or other substance use disorders	Behavioral health administration (BHA) data; Local treatment program data (e.g., methadone programs)
Mental health diagnosis prevalence	% of population with serious mental illness (SMI)	BHA data; SAMHSA homeless programs data
Transition probabilities between housing states*	Weekly or monthly probability of moving from unsheltered to shelter, shelter to transitional housing, transitional to supportive housing, supportive housing to market rental, etc.	Literature estimates (e.g., National homelessness research center); local HMIS longitudinal data; Denver homeless out loud reports
Return to homelessness probability	Probability of returning to homelessness from housing	HUD studies; linked HMIS administrative datasets
Mortality rate	Age-specific mortality rates among homeless populations	Local mortality surveillance; National violent death reporting system; published studies (e.g., homeless mortality meta-analyses)
Service engagement rates*	Probability of accessing services (e.g., shelter, treatment programs, housing navigation) per time step	HMIS service records; BHA service utilization data; Local health department outreach program data
Shelter capacity limits	Number of shelter beds available	Local CoC reports; HMIS shelter data
Supportive housing unit availability	Number of supportive housing units or vouchers	Local housing authority records (e.g., DHA - Denver housing authority); HUD housing inventory count (HIC)
Substance use treatment availability	Slots for methadone, buprenorphine, or behavioral health programs	BHA treatment access data; Local opioid treatment program (OTP) records
Incarceration events	Probability of incarceration/re-entry into homelessness	Local jail and reentry program data; Denver department of public safety reports
Peer network formation probability	Probability of agents forming social ties based on shared characteristics (age, health status, service use)	To be estimated; can be informed by qualitative research or targeted literature (Social networks among unhoused populations)
Weather-related movement effects	Impact of cold weather on transitions (e.g., increased movement to shelters during cold snaps)	NOAA weather data; published studies on weather and shelter utilization
Housing affordability thresholds	Affordable housing cost levels relative to agent income or voucher amount	Local AMI (Area median income) data (Denver office of housing stability); HUD income limit tables

*Parameters that are most uncertain and subject to sensitivity analysis.

### 2.3 Outcomes

#### 2.3.1 Model parameters

As outlined in Table 2, the model outcomes are categorized into housing-related, health, budgetary, and value-based. The modeling will be executed in an open-source programming language. In the agent-based model, each agent will be assigned traits that determine their endogenous features and behavior. The population of agents at model initialization will be representative of the population of interest for a particular analysis (e.g., people who inject drugs) based on city-specific data. Key behaviors to be encoded may include drug use initiation, frequency, and duration, syringe sharing, sexual activity, and unprotected intercourse. Other behaviors to be encoded may include utilizing emergency shelters and propensity for unsheltered living versus in encampments. Overdose can occur in anyone who has the “drug use” trait. We plan to sample the probability of linkage to services from a series of distributions, dependent on each agent’s characteristics and parameterized from data.

**Table 2:** Preliminary model outcomes.

Category	Outcomes
Housing-related	(1) Number of transitions between steps in the continuum, (2) Duration of stay in each step of the continuum, (3) Costs of housing including costs of associated services
Health	(1) Life expectancy, (2) Quality-adjusted life-years (QALYs), (3) HIV incidence and rate, (4) Overdose death incidence and rate, and (5) cause-specific mortality rates
Budgetary	(1) City expenditures, overall and by relevant department/budget component, (2) Total health sector costs, (3) Societal cost
Value-based	(1) Incremental cost-effectiveness ratios, (2) Net monetary benefit

### 2.4 Analysis plan

#### 2.4.1 GMB analyses

Each activity in the GMB sessions generates qualitative information that will be first digitized by the study team. These digital versions will be analyzed in multiple ways. For activities where a diagram was the main output, such as the mapping interactions activity, we will identify which interactions might be modeled and which ones might be simplified through assumptions in the modeling. For the key stakeholder activity, we will develop a graph and identify clusters or categories of keys stakeholders. We will create tables for activities where participants were asked to vote or rank their answers. These digital versions of GMB outputs and their analysis will be validated by GMB workshop participants prior to passing them on to the modelers on the study team for the purposes of model development, calibration, and validation.

#### 2.4.2 Model analyses

First, the study team will simulate interventions for Denver and San Francisco to compare outcomes across all dimensions by randomizing agents to receive the interventions at each point of the cascade and assessing whether the interventions are effective for primary study outcomes (Table 2), and whether there are unintended consequences of implementation. We can simulate randomized trials varying the ‘dose’ of each intervention (e.g., numbers of services provided) and duration. For each intervention the study team will produce effect estimates and generate credible intervals across stochastic model runs. Given that certain outcomes may represent competing risks, depending on the study question, model output may be analyzed further using Fine-Gray models or similar methods. Based on the most effective interventions identified, we will generate projections and data visualizations to inform policy. Potential interventions to be modeled include involuntary displacement with and without housing offers, oral PrEP prescribing to populations in shelters and transitional housing, and expansion of Housing Opportunities for People with AIDS (HOPWA) vouchers.

Second, we will employ sensitivity analysis to determine the extent to which findings may be generalizable to other jurisdictions that may differ by geographic and environmental conditions, epidemiology of diseases, and service availability. We will range core parameters through their feasible ranges to identify those that drive findings and that could differ between jurisdictions.

Third, we will develop an ordered list of priorities for each city. We will employ a standard cost-effectiveness approach, called a “league table,” to define those city-specific priorities. The league table is an ordered list of interventions in ascending order of priority. The highest priority intervention is that which provides the greatest number of life years for every dollar invested in implementation. In addition, we will construct league tables where the benefit being measured is QALYs gained, and HIV infections and overdose averted. Comparing priorities on a cost per life-year, cost per-HIV infection or overdose averted, and QALY basis will demonstrate the roles that mortality and quality of life each play in decision making.

Fourth, we will investigate synergies that are generated when implementing more than one intervention simultaneously (a package of innovations) and assuming a systems-thinking perspective. We will then use sensitivity analysis to develop generalizable messages about priorities.

## 3 Discussion

The iHOUSE model will be the first comprehensive model of the housing continuum for the US. It will be informed by a community engaged research methodology and parameterized using linked local data. We will then use insights from the GMB process and continual conversations with stakeholders to simulate pressing policy questions around how to improve both health and housing outcomes. We acknowledge that there is a wide range of opinions through community engaged research. Unlike other studies, when differences come up in the session, we can account for these differences in the model and how the analyses are done. We expect to have differences in opinions, and a large benefit is that we can account for these expert opinions in various scenarios.

Other models have been designed to answer discrete questions about housing and health outcomes. For example, Stone et al. designed a 58 country-level model to assess unstable housing and HIV and HCV transmission among people who inject drugs (35). While many agent-based models aiming to describe health inequities have focused on individual behavior, models that incorporate socioeconomic environments and social networks may provide more comprehensive representations of social determinants of health aligned with contemporary public health frameworks (36). Comprehensive models like the one we propose may be able to better inform structural public health and policy interventions for greater population-level impact. The use of an agent-based model in particular will enable novel insights from the emergence of population-level phenomena from individual behaviors, as well as subgroup-specific outcomes.

There are several limitations to this study design. First, despite our experience with community engaged research, it is possible that the GMB process and the ultimate structure of the model that is dictated by data availability will not be fully compatible. Nevertheless, the process of community engagement is crucial and can stand alone if this happens. Reporting barriers encountered during the GMB process itself will help develop GMB for the field of agent-based model building. Second, it could be difficult to fit the model to observed data. If so, we will increase the number of simulations as well as investigate the scenarios that have the best and worst likelihood values and potentially constrain certain parameter distributions to allow for convergence. We will consider whether the calibration targets are biased and report on uncertainty. Third, though we have good data sources from primary datasets, randomized trials, and nationally representative cohorts, there is still uncertainty in our data which might skew results. We will plan for robust scenario analyses and deterministic and probabilistic sensitivity analyses to overcome uncertainty. There are also several limitations to the agent-based model, including potentially limited generalizability as the model will be calibrated to data from two cities, and high computational cost.

## 4 Ethics and dissemination

The deliverables will be both community focused and academic. For the community, we will develop a set of white papers for policymakers regarding findings from the barriers and stakeholders exercises as well as outcomes associated with varying intervention levers. We will develop a toolkit for other communities grappling with housing instability and homelessness to replicate this process on their own. We will publish a series of manuscripts that contribute to best practices in GMB for agent-based models. Finally, we plan to develop training curriculum for researchers for broad dissemination.

### 4.1 Data storage, retention and access

In order to maximize the impact of this work, we will comply with all NIH policies and guidance related to data and research resource sharing while protecting study subjects’ rights to privacy and adhering to all appropriate state and federal confidentiality requirements and privacy guidelines (e.g., Health Information Portability and Accountability Act, HIPAA). This includes reporting all results per NIH policy, as well as ensuring that all final peer-reviewed journal manuscripts that arise from these funds are submitted to PubMed Central immediately upon acceptance for publication.

Occasionally, colleagues may request access to model outputs for further analysis or to inform their own modeling work. In such a case, we will work to make those simulation data available to other investigators after discussion and under a formal data-sharing agreement that provides for: (1) commitment to use data for research purposes only; (2) commitment to use appropriate information technology systems to keep data secure; and (3) commitment to returning or destroying data after analyses are complete.

Beyond GitHub and the public team website to share code and analytic resources, the primary mode of sharing results and interpretation of data will be through contributions at scientific meetings and timely publication of scientific accomplishments in peer-reviewed scientific journals, with technical appendices containing input data parameters whenever appropriate. Further, we will work with the departments of communications to disseminate results and publication summaries in non-technical language to ensure reach to a broader audience.

In addition, we will continue to share our findings by serving as advisors and consultants to important medical and patient advocacy groups and policy decision makers.

### 4.2 Dissemination

The proposed agent-based model will serve as a “virtual laboratory” for testing interventions across different environments, each with different socioeconomic conditions, epidemiologic features, resource constraints, and sociopolitical priorities. Our efforts will begin in Denver and San Francisco, which will allow us to validate the model structure. Then, we will employ this model to other settings with either established or developing data structures. We will work with additional municipalities, especially in rural areas, as they construct datasets like those we are using and work to address homelessness locally. This model will be built to evolve alongside the evolving HIV, overdose, and homelessness crises.
